# Supplementary material for: Identification and Expression Analysis of Stilbene Synthase Genes in Arachis hypogaea in Response to Methyl Jasmonate and Salicylic Acid Induction
Source: Plants (Basel). 2022 Jul 5;11(13):1776. doi: 10.3390/plants11131776 (PMC9268999; doi:10.3390/plants11131776)
Supplement: Supplementary file 1 [file plants-11-01776-s001.zip › plants-1725377-supplementary.pdf]

Supplementary Material

Identification and expression analysis of *Stilbene synthase* genes in *Arachis hypogaea* in response to Methyl Jasmonate and Salicylic Acid induction

Zuhra Qayyum[1,+], Fatima Noureen[1,+], Maryam Khan[1], Marrium Khan[1], Ghulam Haider[1], Faiza Munir[1], Alvina Gul[1], Rabia Amir[1*]

[1]Department of Plant Biotechnology, Atta-ur-Rahman School of Applied Biosciences (ASAB), National University of Sciences and Technology (NUST), 44000, Islamabad, Pakistan

*** Correspondence:**
Rabia Amir
rabi.amir@hotmail.com, rabia@asab.nust.edu.pk

1 Supplementary Figures and Tables

1.1 Supplementary Table

Table S1: Nucleotide sequence of the nine contigs found in *Arachis hypogaea*

Sr. No.	Nucleotide Sequences	Contig Results
1	*Arachis hypogaea* mRNA for stilbene synthase, complete CDS	• 20452 Conserved<br>• 31055 Not conserved<br>• 2394 Conserved<br>• 26372 Not conserved<br>• 19158 Not conserved
2	*Arachis hypogaea* clone JC2 putative stilbene synthase gene, complete cds	• 20452 Conserved<br>• 31055 Not conserved<br>• 2394 Conserved<br>• 19158 Not conserved
3	*Arachis hypogaea* cultivar Champyeong stilbene synthase (STS) mRNA, complete cds	• 20452 Conserved<br>• 31055 Not conserved<br>• 2394 Conserved<br>• 26372 Not conserved
4	*Arachis hypogaea* clone 05_08E putative stilbene synthase mRNA, partial cds	• 20452 Conserved<br>• 31055 Not conserved<br>• 2394 Conserved<br>• 26372 Not conserved
5	*Arachis hypogaea* clone 04_07D putative stilbene synthase mRNA, partial cds	• 20452 Conserved<br>• 31055 Not conserved<br>• 2394 Conserved<br>• 26372 Not conserved

6	*Arachis hypogaea* clone 03_11D putative stilbene synthase mRNA, partial cds	• 20452 Conserved<br>• 31055 Not conserved<br>• 2394 Conserved<br>• 26372 Not conserved
7	*Arachis hypogaea* stilbene synthase mRNA, complete cds	• 20452 Conserved<br>• 31055 Not conserved<br>• 2394 Conserved<br>• 26372 Not conserved
8	UNVERIFIED: *Arachis hypogaea* clone 04_06E putative stilbene synthase-like mRNA, partial sequence	• 20452 Conserved<br>• 31055 Not conserved<br>• 2394 Conserved<br>• 26372 Not conserved
9	*A.hypogaea* gene for stilbene synthase	• 20452 Conserved<br>• 31055 Not conserved<br>• 2394 Conserved<br>• 26372 Not conserved

Table S2. Attributes of five *Stilbene synthase* (*AhSTS*s) genes identified in peanut.

**Genes**	**NCBI Accession No.**	**Nucleotide Sequence Length**	**Length of coding sequence (position of CDS)**	**Length of amino acid sequence**
		**Bp**	**Bp**	**Aa**
AhSTS1	MT663407	1208 bp	1169 (7-1176)	389
AhSTS2	MT710308	1454 bp	1169 (82-1251)	389
AhSTS3	MT710309	1437 bp	1169 (50-1219)	389
AhSTS4	MT710310	1548 bp	869 (72-941)	289
AhSTS6	MT710312	1571 bp	1169 (81-1250)	389

Table S3. Physiochemical properties of *Stilbene synthase* (*AhSTS*s) genes identified in peanut.

**Gene ID**	**[a]aa**	**[b]pI**	**[c]Mw**	**[d]II**	**[e]AI**	**[f]EC**	**[g]GRAVY**	**Half life**	**Sub-cellular localization**
AhSTS1	389	5.96	42952.7	35.03	91.23	33390	-0.088	>10 hours	cyto
AhSTS2	389	6.84	59343.75	40.66	93.70	54360	-0.070	>10 hours	cyto
AhSTS3	389	5.66	42840.39	35.17	91.98	33390	-0.078	>10 hours	cyto
AhSTS4	289	6.71	32125.13	33.36	90.42	19285	-0.107	>10 hours	cyto
AhSTS6	389	5.56	42817.30	32.02	92.01	34755	-0.084	>10 hours	cyto

[a]Amino acid (protein length); [b]Isoelectric point; [c]Molecular weight (Da); [d]Instability index; [e]Aliphatic Index; [f]Extinction co-efficient; [g]Grand average of hydrophobicity.

Table S4: Cis-acting elements in the promoter region of stilbene synthase (*AhSTSs)* genes

Gene ID	Name	Number	sequence	Function
AhSTS1	Box 4	3	ATTAAT	part of a conserved DNA module involved in light responsiveness
	CAAT-Box	26	CAAAT/CAAT	common cis-acting element in promoter and enhancer regions
	GATA-motif	1	AAGGATAAGG	part of a light responsive element
	LAMP-element	1	CTTTATCA	part of a light responsive element
	MRE	1	AACCTAA	MYB binding site involved in light responsiveness
	TATC-box	1	TATCCCA	cis-acting element involved in gibberellin-responsiveness
	TCA-element	1	CCATCTTTTT	cis-acting element involved in salicylic acid responsiveness
	TGA-element	1	AACGAC	auxin-responsive element
	chs-CMA1a	1	TTACTTAA	part of a light responsive element
AhSTS2	ABRE	3	ACGTG/CACGTG	cis-acting element involved in the abscisic acid responsiveness
	ARE	1	AAACCA	cis-acting regulatory element essential for the anaerobic induction
	Box 4	2	ATTAAT	part of a conserved DNA module involved in light responsiveness
	CAAT-box	30	CAAAT/CAAT/CCAAT	common cis-acting element in promoter and enhancer regions
	CGTCA-motif	3	CGTCA	cis-acting regulatory element involved in the MeJA-responsiveness
	G-Box	2	CACGTT/CACGTG	cis-acting regulatory element involved in light responsiveness
	G-box	1	CACGTG	cis-acting regulatory element involved in light responsiveness
	TCT-motif	1	TCTTAC	part of a light responsive element
	TGA-element	1	AACGAC	auxin-responsive element

	TGACG-motif	3	TGACG	cis-acting regulatory element involved in the MeJA-responsiveness
	chs-Unit 1 m1	1	ACCTAACCCGC	part of a light responsive element
AhSTS3	ABRE	2	CACGTG/ACGTG	cis-acting element involved in the abscisic acid responsiveness
	Box 4	6	ATTAAT	part of a conserved DNA module involved in light responsiveness
	CAAT-box	25	CAAAT/CAAT	common cis-acting element in promoter and enhancer regions
	CGTCA-motif	1	CGTCA	cis-acting regulatory element involved in the MeJA-responsiveness
	G-Box	1	CACGTG	cis-acting regulatory element involved in light responsiveness
	G-box	1	CACGTG	cis-acting regulatory element involved in light responsiveness
	GATA-motif	1	AAGATAAGATT	part of a light responsive element
	I-box	1	atGATAAGGTC	part of a light responsive element
	RY-element	1	CATGCATG	cis-acting regulatory element involved in seed-specific regulation
	TCA-element	1	CCATCTTTTT	cis-acting element involved in salicylic acid responsiveness
	TCT-motif	1	TCTTAC	part of a light responsive element
	TGACG-motif	1	TGACG	cis-acting regulatory element involved in the MeJA-responsiveness
AhSTS4	ABRE	2	CACGTG/ACGTG	is-acting element involved in the abscisic acid responsiveness
	Box 4	6	ATTAAT	part of a conserved DNA module involved in light responsiveness
	CAAT-box	26	CAAAT/CAAT	common cis-acting element in promoter and enhancer regions
	CGTCA-motif	1	CGTCA	cis-acting regulatory element involved in the MeJA-responsiveness
	G-Box	1	CACGTG	cis-acting regulatory element involved in light responsiveness
	G-box	1	CACGTG	cis-acting regulatory element involved in light responsiveness
	GATA-motif	1	AAGATAAGATT	part of a light responsive element

	I-box	1	atGATAAGGTC	part of a light responsive element
	RY-element	1	CATGCATG	cis-acting regulatory element involved in seed-specific regulation
	TCA-element	1	CCATCTTTTT	cis-acting element involved in salicylic acid responsiveness
	TCT-motif	1	TCTTAC	part of a light responsive element
	TGACG-motif	1	TGACG	cis-acting regulatory element involved in the MeJA-responsiveness
AhSTS6	ABRE	2	CACGTG/ACGTG	cis-acting element involved in the abscisic acid responsiveness
	Box 4	6	ATTAAT	part of a conserved DNA module involved in light responsiveness
	CAAT-box	27	CAAAT/CAAT	common cis-acting element in promoter and enhancer regions
	CGTCA-motif	1	CGTCA	cis-acting regulatory element involved in the MeJA-responsiveness
	G-Box	1	CACGTG	cis-acting regulatory element involved in light responsiveness
	G-box	1	CACGTG	cis-acting regulatory element involved in light responsiveness
	GATA-motif	1	AAGATAAGATT	part of a light responsive element
	I-box	1	atGATAAGGTC	part of a light responsive element
	RY-element	1	CATGCATG	cis-acting regulatory element involved in seed-specific regulation
	TCA-element	1	CCATCTTTTT	cis-acting element involved in salicylic acid responsiveness
	TCT-motif	1	TCTTAC	part of a light responsive element
	TGACG-motif	1	TGACG	cis-acting regulatory element involved in the MeJA-responsiveness

Table S5. Primers identity and sequences used for Stilbene synthase (*AhSTSs*) gene amplification and RT-PCR analysis

Primer Name	Purpose of primer	5’-Forward	3’-Reverse
AhSTS1	Full length gene amplification	AGCGAAGCTTTACTCTCA	ATGGGCCAATCTAGACCT
	RT-PCR	ACCAGGTTGAACAGAAGGTG	ACCCCAATCAAATCCTTCTC
AhSTS2	Full length gene amplification	TGATCTTCTGCACCACAAGC	CTCAGAACAAACCACCAGCA
	RT-PCR	CACGTGATGTTCTTAGCGAT	TTCAATAGTGAGGCCAGGTC
AhSTS3	Full length gene amplification	TTTCAGCGCATTTGTGAAAG	CTTGGTTGCTGCCTCTTTTC
	RT-PCR	TCACACATTTGATCTTCTGC	TGTTATTTTCAGCCAAGTCC
AhSTS4	Full length gene amplification	TTTCAGCGCATTTGTGAGAG	AGTTGCAGCCTCTTTTCCAA
	RT-PCR	AGCACATACGCAGATTACTA	CAAGGACGGTGCTTTATATG
AhSTS6	Full length gene amplification	TTTCAGCGCATTTGTGAGAG	AGTTGCAGCCTCTTTTCCAA
	RT-PCR	TGGTTCTGATCCTATTCCAG	AAAGCTTTAGTGAGTGCATC
Actin	Reference gene	CAGGCCGTTCTCTCCCTTTAT	CATCAAGGCATCGGTGAGAT

Table S6. Correlation between time and the expression of *Stilbene synthase* (*AhSTS*s) genes under the treatment of methyl jasmonate and salicylic acid in peanut.

Genes		Methyl Jasmonate	Salicylic Acid
		**Bard-479**	**Bard-479**
AhSTS1	*R*	0.8905	-0.4962
	p-value	0.0428	0.3952
AhSTS 2	*R*	0.7479	-0.5012
	p-value	0.1461	0.3897
AhSTS 3	*R*	0.2974	-0.4976
	p-value	0.6269	0.3936
AhSTS 4	*R*	0.9622	0.06101
	p-value	0.0088	0.9224
AhSTS 6	*R*	-0.3062	0.05057
	p-value	0.6163	0.9356

p-value (0.05) is considered to be significant; *r* is representing Pearson's correlation coefficient.

Table S7. Correlation among the expression of *Stilbene synthase* (*AhSTS*s) genes in peanut.

BARD-479		Methyl Jasmonate				Salicylic Acid			
		AhSTS2	*AhSTS3*	*AhSTS4*	*AhSTS6*	*AhSTS2*	*AhSTS3*	*AhSTS4*	*AhSTS6*
AhSTS1	*R*	0.6679	0.5668	0.7984	-0.2193	0.07160	0.2334	-0.5149	-0.5254
	p-value	0.2180	0.3191	0.1053	0.7231	0.9089	0.7056	0.3747	0.3632
AhSTS2	*R*	----	0.6804	0.7972	0.3949	----	0.9795	-0.3195	-0.3079
	p-value	---	0.2062	0.1062	0.5105	---	0.0035	0.6002	0.6142
AhSTS3	*R*	---	---	0.2755	0.6279	---	---	-0.3881	-0.3808
	p-value	----	----	0.6537	0.2567	----	---	0.5185	0.5271
AhSTS4	*R*	---	---	---	-0.1779	---	---	---	0.9997
	p-value	---	---	---	0.7747	---	---	---	< 0.0001

p-value (0.05) is considered to be significant; *r* is representing Pearson's correlation coefficient.

1.2 Supplementary Figures

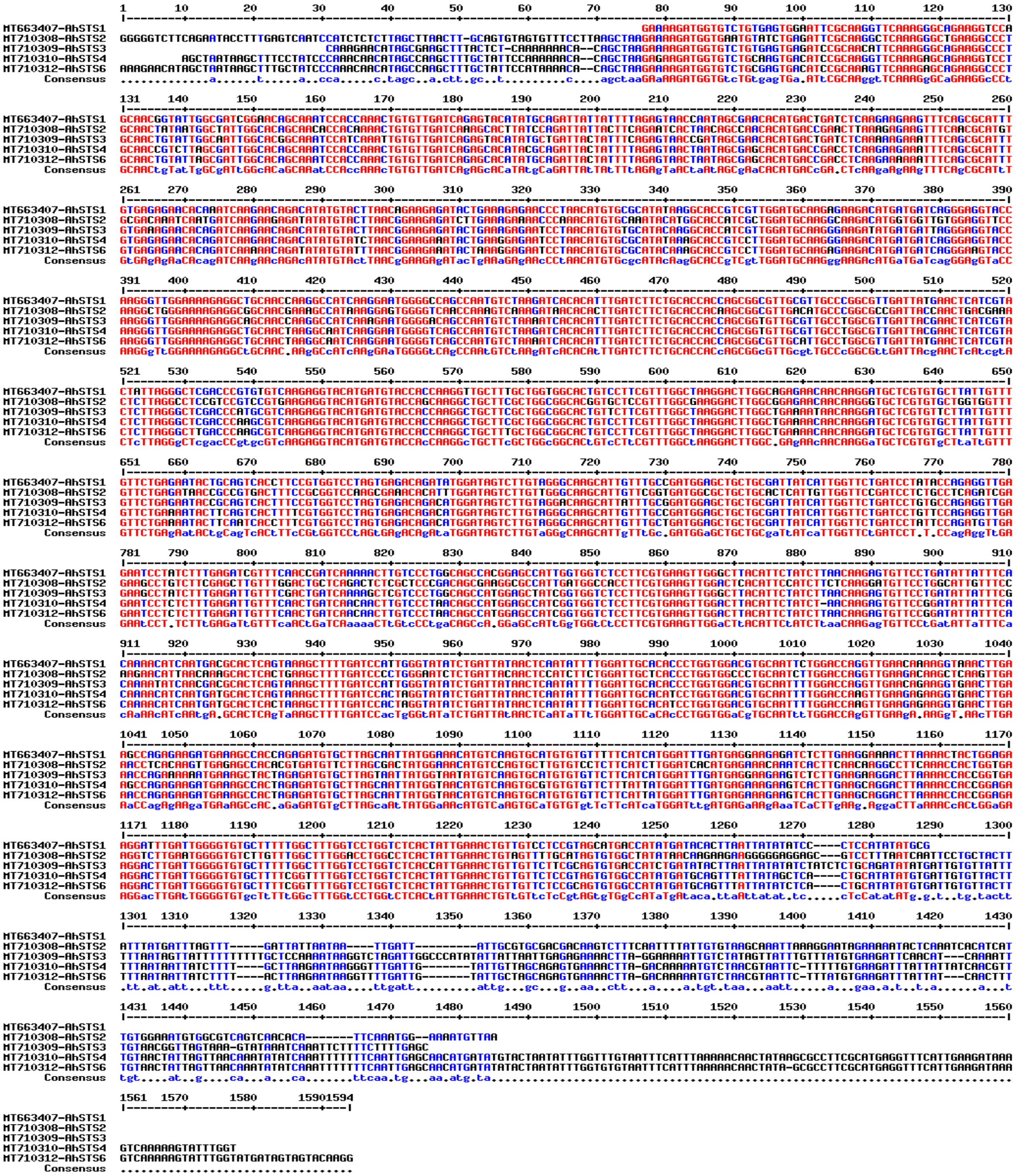

Figure S1. Multiple sequence alignment of nucleotide sequence of the *AhSTS1*, *AhSTS2*, *AhSTS3*, *AhSTS4*, *AhSTS6.* The sequences conserved across all genes are highlighted in red while non-conserved sequences are shown in black. The sequences highlighted in blue shows sequences conserved in some genes.

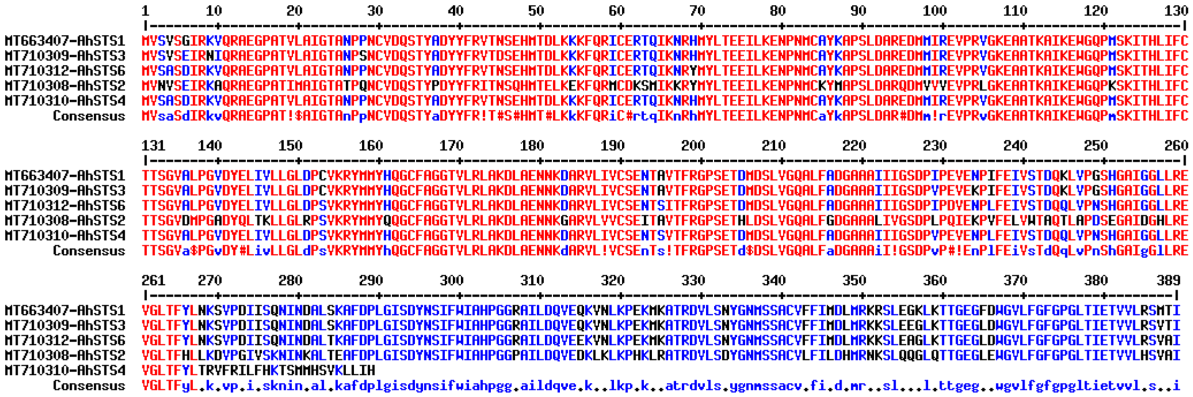

Figure S2. Multiple sequence alignment of amino acid sequences of *AhSTS1*, *AhSTS2*, *AhSTS3*, *AhSTS4*, and *AhSTS6*. The sequences conserved across all genes are highlighted in red while non-conserved sequences are shown in black. The sequences highlighted in blue shows sequences conserved in some genes.

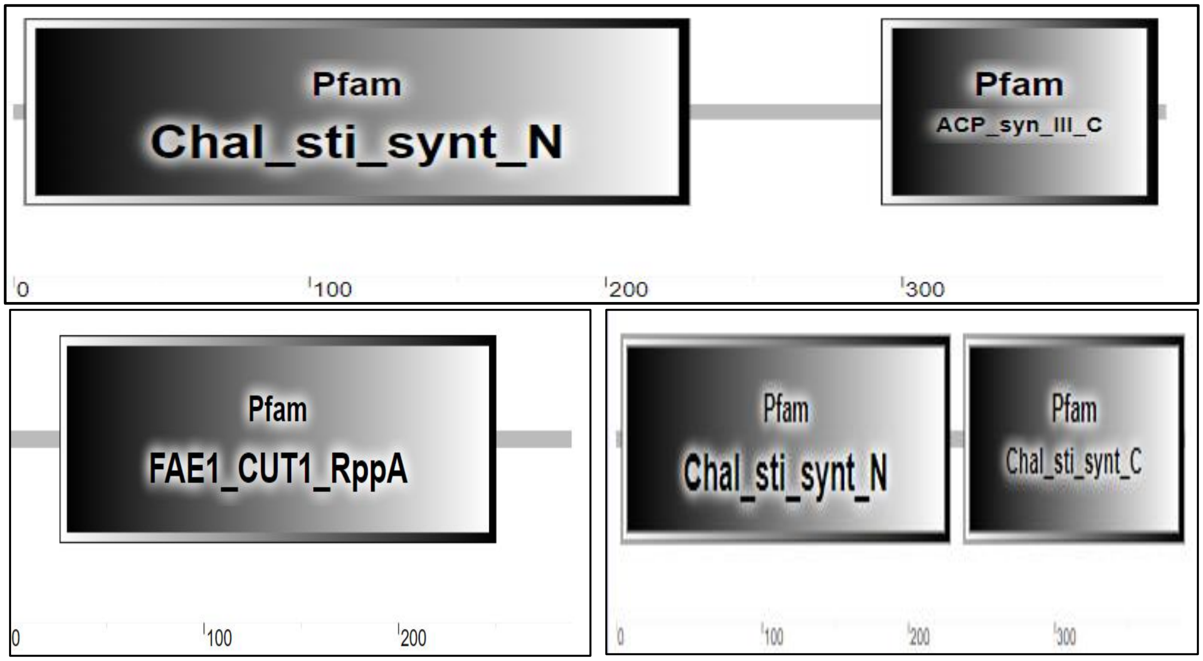

Figure S3. Showing the conserved N-terminal Chal_sti_synt domain and C-terminal ACP_syn_III FAE1_CUT1_rppA, in the sequences of *AhSTS1*, *AhSTS2*, *AhSTS3*, *AhSTS4* and *AhSTS6*.

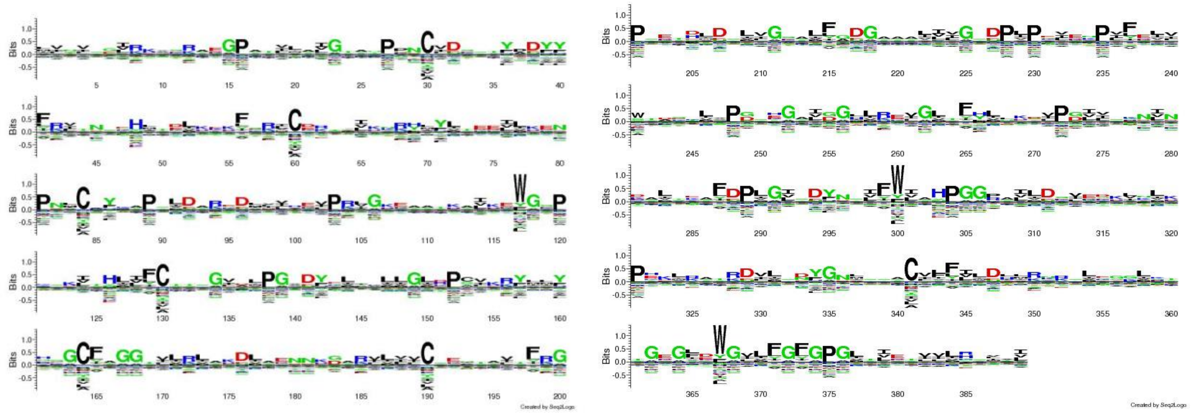

Figure S4. Sequence logo of *AhSTS*s. X-axis showing the position of position of amino acid whereas stack of symbols representing the amino acids (big stacks represent conserved positions and small stacks represents variable position).

Predicted Secondary Structure of *Arachis hypoggaea*

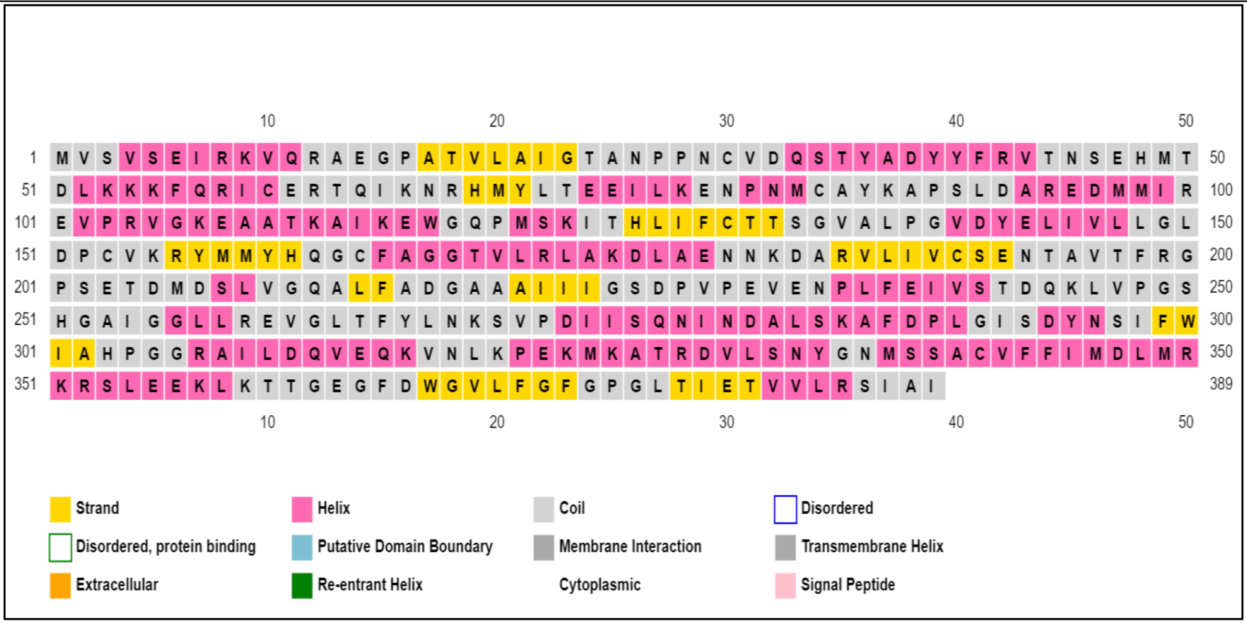

Figure S5: secondary structure of *Arachis hypogaea STS1* showing alpha helices and beta sheets. Loops are also observed in the structure.

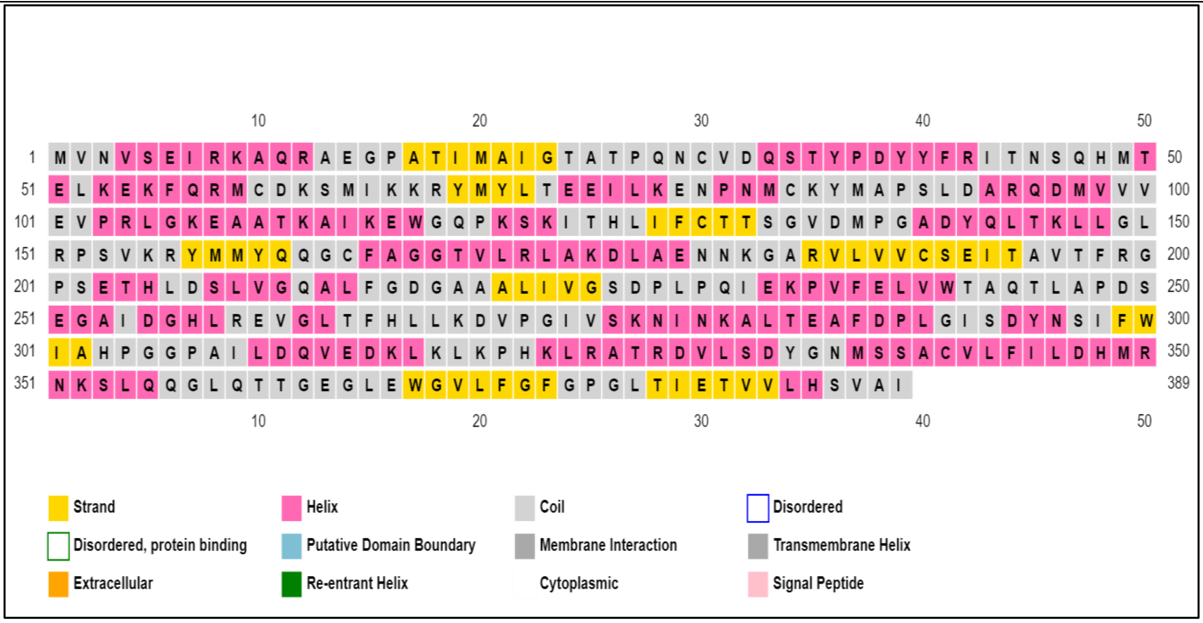

Figure S6: Predicted secondary structure of *Arachis hypogaea STS2* showing alpha helices and beta sheets. Loops are also observed in the structure.

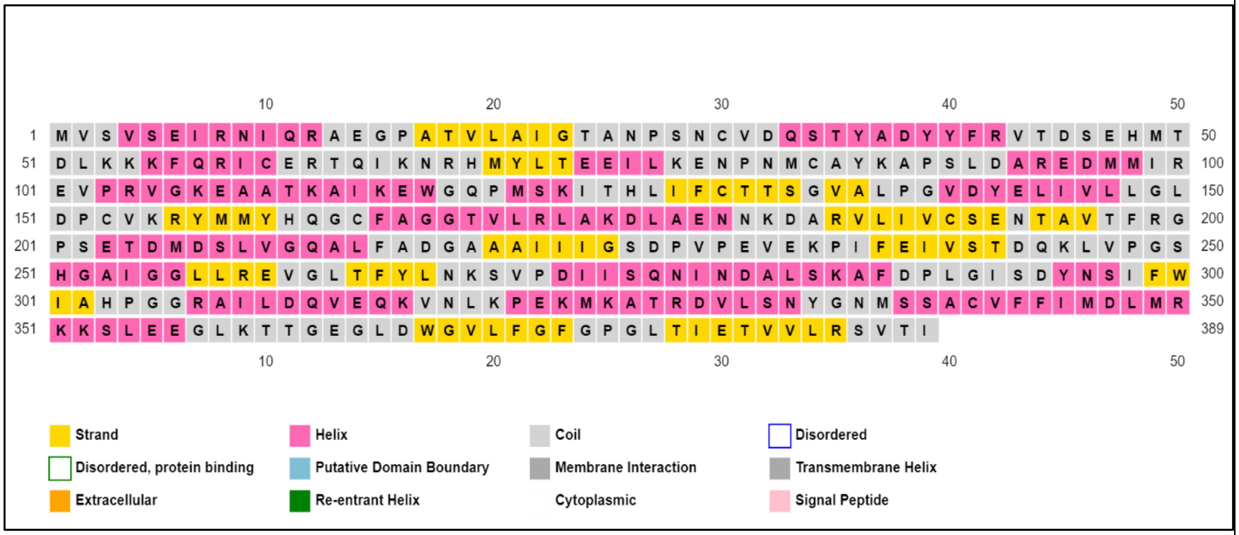

Figure S7: Predicted secondary structure of *Arachis hypogaea STS3* showing alpha helices and beta sheets. Loops are also observed in the structure.

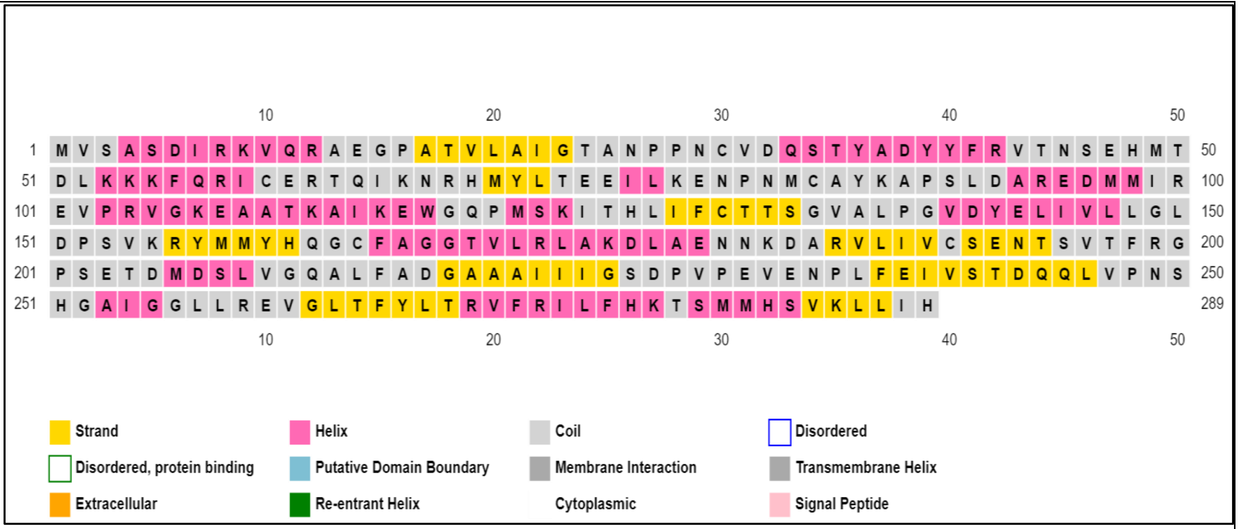

Figure S8: Predicted secondary structure of *Arachis hypogaea STS4* showing alpha helices and beta sheets. Loops are also observed in the structure.

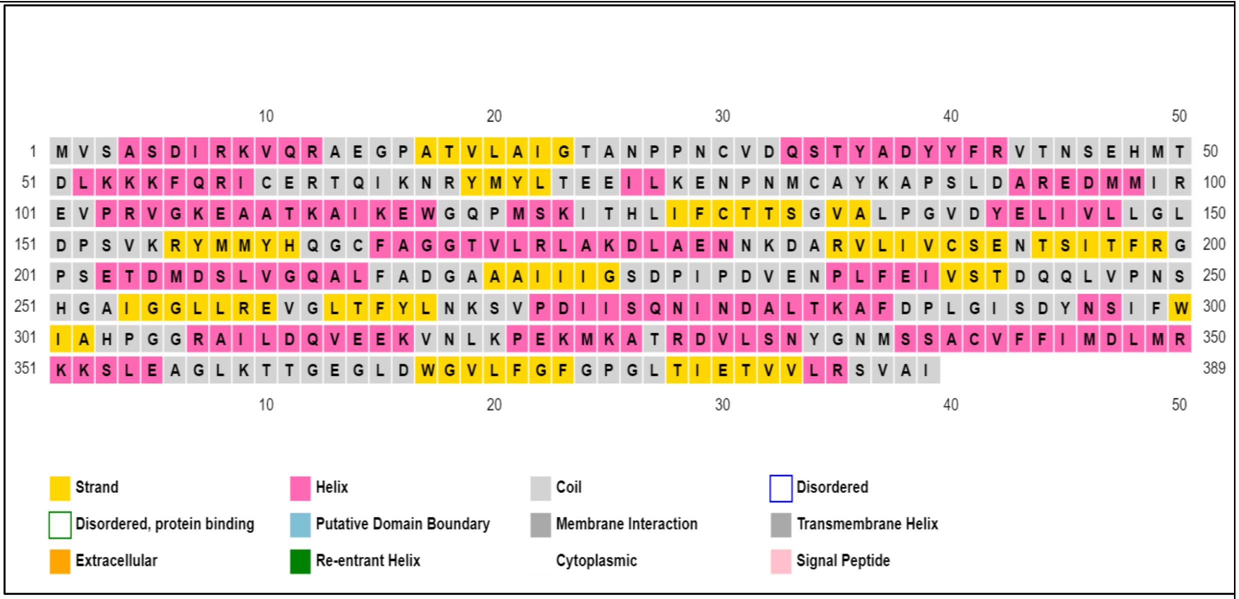

Figure S9: Predicted secondary structure of *Arachis hypogaea STS6* showing alpha helices and beta sheets. Loops are also observed in the structure.

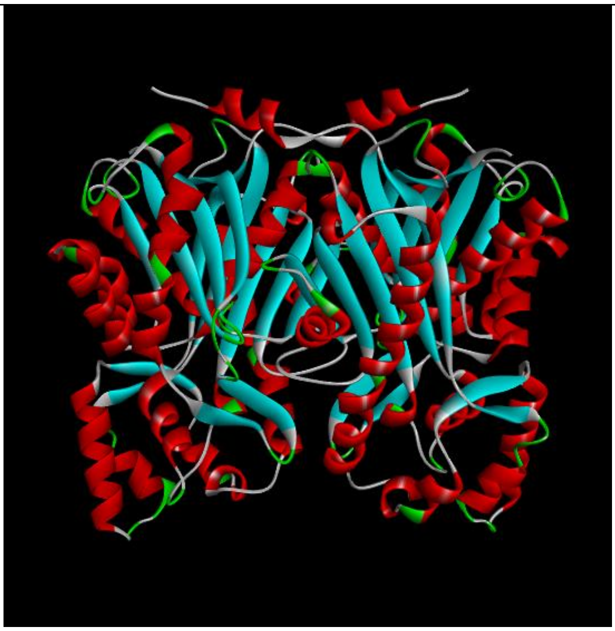

Figure S10. 3D structure of *AhSTS*1predicted from the SWISS-MODEL. The red colour indicates helices, green colour represents loops and cyan colour represent β-sheets.

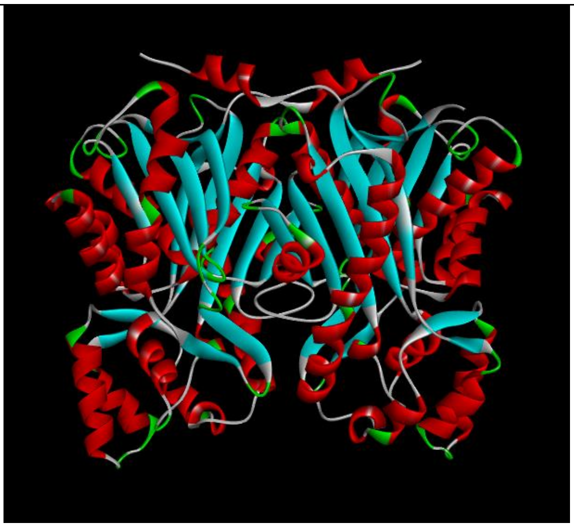

Figure S11. 3D structure of *AhSTS2* predicted from the SWISS-MODEL. The red colour indicates helices, green colour represents loops and cyan colour represent β-sheets.

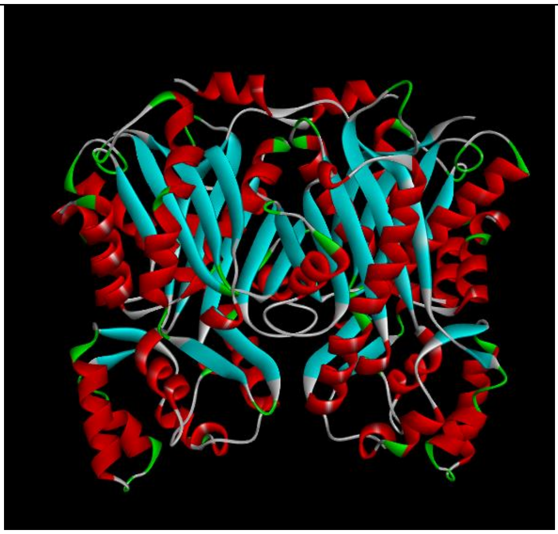

Figure S12. 3D structure of *AhSTS3* predicted from the SWISS-MODEL. The red colour indicates helices, green colour represents loops and cyan colour represent β-sheets.

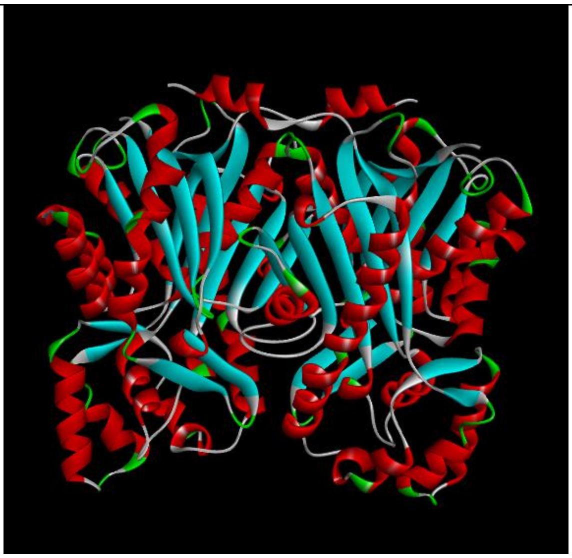

Figure S13. 3D structure of *AhSTS4* predicted from the SWISS-MODEL. The red colour indicates helices, green colour represents loops and cyan colour represent β-sheets.

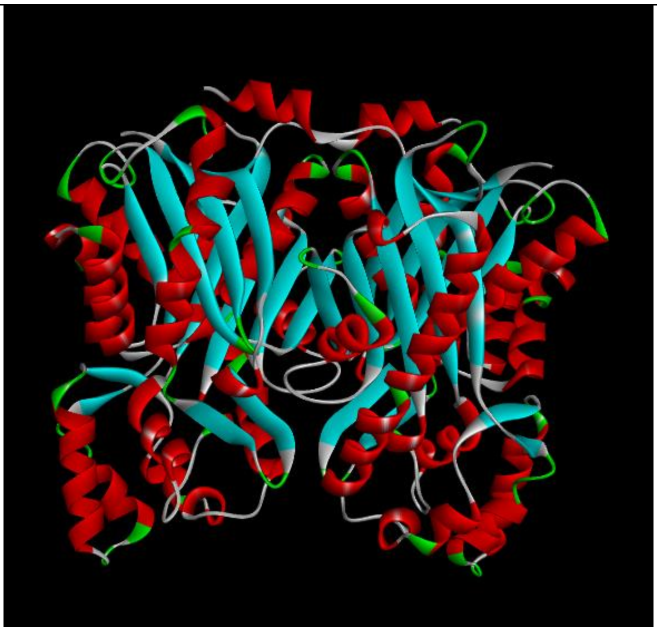

Figure S14. 3D structure of *AhSTS6* predicted from the SWISS-MODEL. The red colour indicates helices, green colour represents loops and cyan colour represent β-sheets.

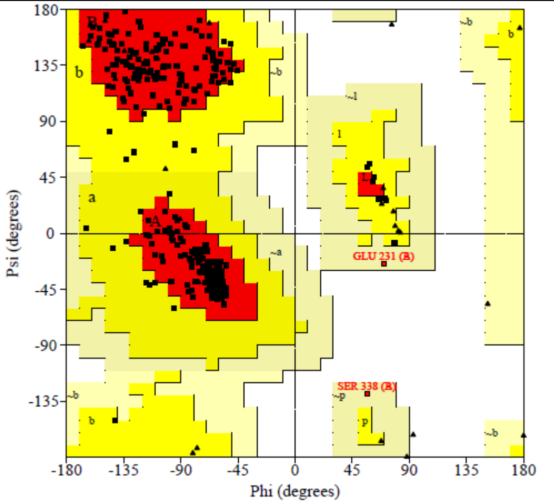

Figure S15. Ramachandran Plot of *stilbene synthase* protein in *Arachis hypogaea.* The graph shows the location of amino acid residues in the favored region of protein. For *AhSTS1*, the location of amino acids in the favored region 92.9% forming a stable protein.

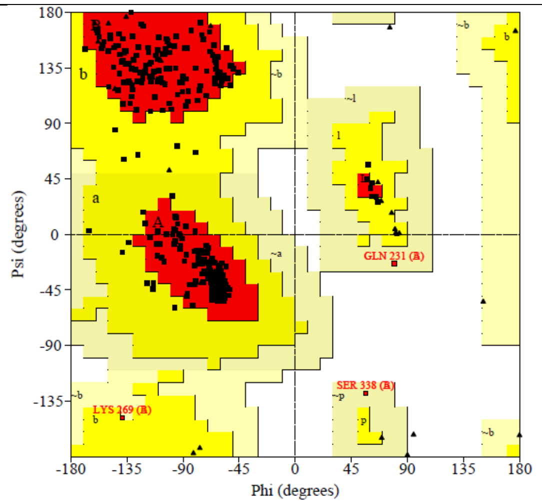

Figure S16. Ramachandran Plot of *stilbene synthase* protein in *Arachis hypogaea.* The graph shows the location of amino acid residues in the favored region of protein. For *AhSTS2*, the location of amino acids in the favored region 93.8% forming a stable protein.

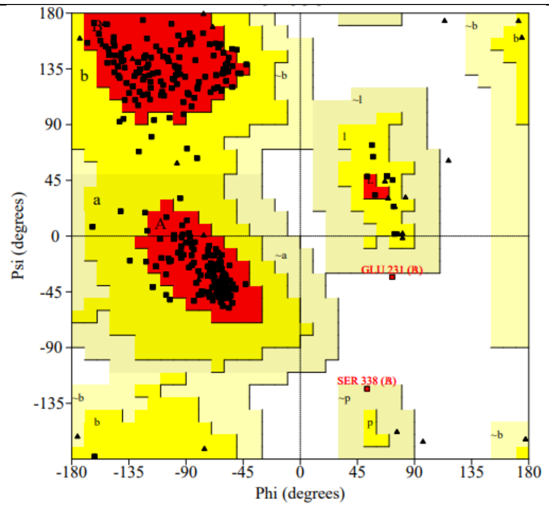

Figure S17. Ramachandran Plot of *stilbene synthase* protein in *Arachis hypogaea.* The graph shows the location of amino acid residues in the favored region of protein. For *AhSTS3*, the location of amino acids in the favored region 92 % forming a stable protein.

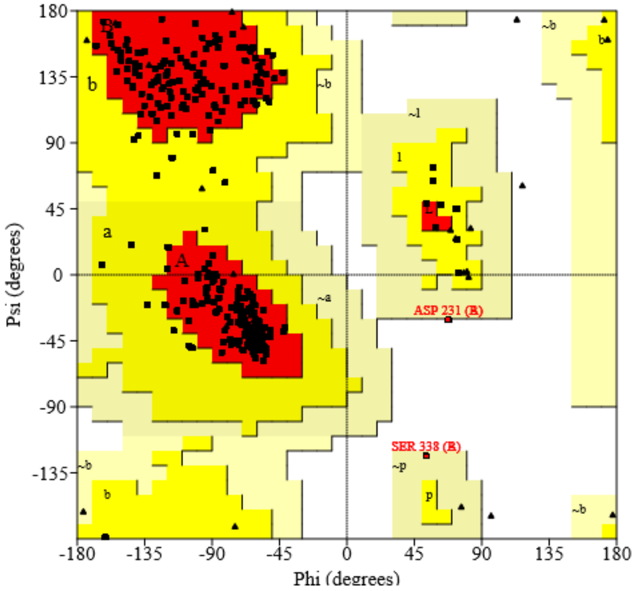

Figure S18. Ramachandran Plot of *stilbene synthase* protein in *Arachis hypogaea*. The graph shows the location of amino acid residues in the favored region of protein. For *AhSTS4*, the location of amino acids in the favored region 92.3 % forming a stable protein.

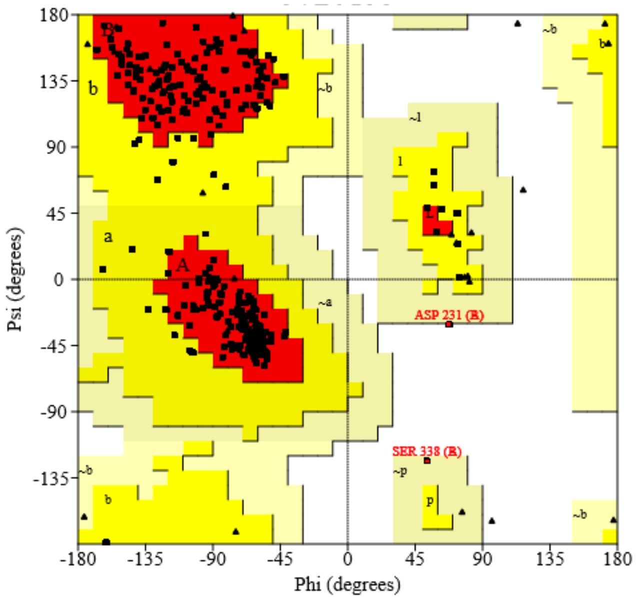

Figure S19. Ramachandran Plot of *stilbene synthase* protein in *Arachis hypogaea*. The graph shows the location of amino acid residues in the favored region of protein. For *AhSTS6*, the location of amino acids in the favored region 92.3 % forming a stable protein.

2. Hormonal Stock Solution 100µM MeJA

Density of MeJA = 1.03g/cm^3

To find molarity of solution

MeJA in solution = 1.03 x 1000

= 1030g/l

Molarity= given mass /Molar mass

= 1030/244.3

= 4.5M

To prepare 150ml stock solution of 100µM MeJA

M1V1 = M2V2

4.5 x 10^6 x V1 = 100 x 150

V1 = 15000/4500000

V1 = 0.0033 ml x 1000

V1 = 3.3 µl MeJA

Procedure:

1. Take 100 ml of distilled water in a sterilized beaker.
2. Place the beaker on hot plate. Pre-warm the distilled water. Add 3.3µl of MeJA to the water and stir the solution. Add absolute ethanol dropwise to dissolve MeJA. Keep on stirring the solution.
3. As soon as MeJA dissolves in dH2O an insoluble transparent layer disappears. Make the volume up to 150 ml by adding warm distilled water. Place the stock solution in transparent bottle wrapped in aluminum foil in refrigerator.
4. Do not add too much alcohol as it will disturb the volume concentration.

Hormonal Stock Solution 1mM SA

1M SA is prepared by adding 138.121 g powdered SA in 1 litre water.

1mM SA is prepared by adding 138.1 mg powdered SA in 1 litre water.

To prepare 200 ml 1mM SA, 27.62mg of SA is dissolved in 200ml dH2O.

Procedure:

- Take a clean beaker and fill it with 150 ml distilled water.
- Place the beaker on hot plate and put magnetic stirrer in the beaker.
- Pre-warm dH2O and add SA pinch by pinch.

- Keep the magnetic stirrer on.
- A white foam is formed.
- Start adding absolute ethanol drop wise in the solution.
- SA starts dissolving. Keep the solution stirred to get smooth consistency.
- When a clear SA solution is formed add dH2O to make up the volume to 200.

Arachis hypogaea Stilbene synthase (AhSTS1) complete CDS

ATGGTGTCTGTGAGTGAGATTCGCAAGGTTCAAAGGGCAGAAGGTCCAGCAACTGTATTGGCGATTGG
AACAGCAAATCCACCAAACTGTGTTGATCAGAGTACATATGCAGATTATTATTTTAGAGTAACTAACAGC
GAACACATGACTGATCTCAAGAAGAAGTTTCAGCGCATTTGTGAGAGAACACAAATCAAGAACAGACAT
ATGTACTTAACAGAAGAGATACTGAAAGAGAACCCTAACATGTGCGCATACAAGGCACCGTCGTTGGAT
GCAAGAGAAGACATGATGATCAGGGAGGTACCAAGGGTTGGAAAAGAGGCTGCAACCAAGGCCATCA
AAGAATGGGGCCAGCCAATGTCTAAGATCACGCATTTGATCTTCTGTACCACCAGCGGCGTTGCGTTGC
CTGGCGTTGATTATGAACTCATCGTACTCTTAGGGCTCGACCCATGCGTCAAGAGGTACATGATGTACCA
CCAAGGCTGCTTCGCTGGTGGCACTGTCCTTCGTTTGGCTAAGGACTTGGCTGAAAACAACAAGGATGC
TCGTGTGCTTATCGTTTGTTCTGAGAATACCGCAGTCACTTTCCGTGGTCCTAGTGAGACAGACATGGAT
AGTCTTGTAGGGCAAGCATTGTTTGCCGATGGAGCTGCTGCGATTATCATTGGTTCTGATCCTGTTCCAG
AGGTTGAGAATCCTCTCTTTGAGATTGTTTCAACTGATCAAAAACTTGTCCCTGGCAGCCATGGAGCCAT
CGGTGGTCTCCTTCGTGAAGTTGGGCTTACATTCTATCTTAACAAGAGTGTTCCTGATATTATTTCACAAA
ACATCAATGATGCACTCAGTAAAGCTTTTGATCCATTGGGTATATCTGATTATAACTCAATATTTTGGATT
GCACACCCTGGTGGACGTGCAATTTTGGACCAGGTTGAACAGAAGGTGAACTTGAAACCAGAGAAGAT
GAAAGCCACCAGAGATGTGCTTAGCAATTATGGTAACATGTCAAGTGCATGTGTGTTTTTCATCATGGAT
TTGATGAGGAAGAGATCTCTTGAAGAAAAACTTAAAACTACTGGAGAAGGATTTGATTGGGGTGTGCTT
TTTGGCTTTGGTCCTGGTCTCACTATTGAAACTGTTGTGCTCCGCAGCATAGCCATATGA

Putative promoter region *AhSTS1*

CATTTGTAAAGCTTGATTGCTCTGTGTGTTCTCTCTAAAAATAAGTTCATTCCTGCAC
TT
CCATGTTGTATTGCAGGTTGGTATGAAGGTAATGGACTATAGGTTTCCATTTTGATTT
GG
CGAGACTTTTTTCCAAGTTTGTCTTTATATTTGTGGATGTTTTTTTTTTTTATGTTTTG
G
ATTTTTTTTAGGTTTTGGATGTTTTATTTTAAAATAATTTTGGATTTTCTTTTTAATGT
T
TCTAAATATTTTTTAATTATTTTTTTATGTTCTTTTTCAATAATTTTAAACGTTAAAAA
A
AACGTTTCTTTTGTTAGGATTTAGATATTTGTTTTTGTTAAATTTTGAAACTTTTTAAT
G
ATTTTAGATTATTTTTATTAGATTTTGAATTTTTTATTATAAAATTAACTAGTTTATTA
A
AAAGTTGGCTAAAGAAGTATCAGTTACCTAGATTTTTTTCTATTCTCATATATGATAG
AA
AAACAGAAATGAACAAATCAATTAGAATTCTAAATATGGCTTCCTATTTATATCTAC
AAC
TAACTAGCTCAGCTAGGCTTAATTTAAGTAATAAATTATATATTTATTATATCTAATA
TT

ATTCAATTATTTTAACAATAATTAAAAATAAGAATAAGATAATTAATAATTAATAAA
TAT
AAAATAAAATAATTTTAAATTGATTTAGACTAATTTTCTATTACCTCCTTATCATTTT
GA
ATATACATAACCTTATTCTGCTTCTTGTGTTATTTTATAAAAAGAAGTAAATGAATTG
TT
AATATGCTTGCTAGAGATGCTTCTTATTAAAAGTGCAGATTGCGATCCAAATCAGGT
CAT
TGCATCATTAGTCTCTCTAAATTGAATTACGAAATAATATTCATATTTTATAAAATGA
AA
AAAATTCCTTTTTTTTATATATATATTTCTTTTCATATGCTAGTCTCATTGCCCGTCTT
T
GCCTCTTTATCATTAGTGTCGTTGCCACCACTCCACCATAACCACTGGCAGCAACAA
GAA
GAAAGTGTGGAGGAAGTGGTAGCGGCATTGCGAATTAAGATGACATGAATATTTTT
TTTA
TATAAAATTAATTTTATTATATTCAACCTTGTATAATAAATTAAAAATTAGACAAGA
AAG
TAACATTATTATATAGTTATGGGATACCACACAATAATATAAAAATTACAAACATAA
TAC
TATCAAGCCTTGCACTGTTGTACCTAATTCTTTTTAGTTTATCTTAAATGGAACTTCA
TA
TGAAGGTGTTTGTAGTTGTCCTAAAATGAAAATGCAACCCAAATATTAGTATGTGTC
ATA
TTGCTCAATCGAAAAGAATTTTATTTATTTTTTACTAACAATTACAAACTTTGAAATT
AA
ATCTTCACATAAAGAAATGACTTTAGACATTTTTGTATAAGTTTTCACTCATCTAATA
AT
ATATAGGCCAATTAGGACCTTATTTCGGAACAAAATAAAATTATTAAAAAATAACA
AAAT
C
Web link
file:///C:/Users/Lenovo%20330/AppData/Local/Temp/Temp1_PlantCARE_8915_Ahsts1_plantCARE.tar.zip/PlantCARE_8915_plantcare.html

Arachis hypogaea Stilbene synthase (AhSTS2) complete CDS

ATGGTGAATGTATCTGAGATTCGCAAGGCTCAAAGGGCTGAAGGCCCTGCAACTAT
AATGGCTATTGGCACAGCAACACCACAAAACTGTGTTGATCAAAGCACTTATCCAG
ATTATTACTTCAGAATCACTAACAGCCAACACATGACCGAACTTAAAGAGAAGTTTC
AACGCATGTGCGACAAATCAATGATCAAGAAGAGATATATGTACTTAACGGAAGAG
ATCTTGAAAGAAAACCCAAACATGTGCAAATACATGGCACCATCGCTGGATGCAAG
GCAAGACATGGTGGTTGTGGAGGTTCCAAGGCTGGGAAAAGAGGCGGCAACGAAA
GCCATAAAGGAGTGGGGTCAACCAAAGTCAAAGATAACACACTTGATCTTCTGCAC
CACAAGCGGCGTTGACATGCCCGGCGCCGATTACCAACTGACGAAACTCTTAGGCC
TCCGTCCGTCCGTGAAGAGGTACATGATGTACCAGCAAGGCTGCTTCGCTGGCGGCA
CGGTGCTCCGTTTGGCGAAGGACTTGGCGGAGAACAACAAGGGTGCTCGTGTGCTG

GTGGTTTGTTCTGAGATAACCGCCGTGACTTTCCGCGGTCCAAGCGAAACACATTTG
GATAGTCTTGTTGGGCAAGCATTGTTCGGTGATGGCGCTGCTGCACTCATTGTTGGTT
CCGATCCTCTGCCTCAGATCGAGAAGCCTGTCTTCGAGCTTGTTTGGACTGCTCAGA
CTCTCGCTCCCGACAGCGAAGGCGCCATTGATGGCCACCTTCGTGAAGTTGGACTCA
CATTCCATCTTCTCAAGGATGTTCCTGGCATTGTTTCCAAGAACATTAACAAAGCAC
TCACTGAAGCTTTTGATCCCCTGGGAATCTCTGATTACAACTCCATCTTCTGGATTGC
TCACCCTGGTGGCCCTGCAATCTTGGACCAGGTTGAAGACAAGCTCAAGTTGAAACC
TCACAAGTTGAGAGCCACACGTGATGTTCTTAGCGACTATGGAAACATGTCCAGTGC
TTGTGTCCTCTTCATCTTGGATCACATGAGAAACAAATCACTTCAACAAGGCCTTCA
AACCACTGGTGAAGGTCTTGAATGGGGTGTCTTGTTTGGCTTTGGACCTGGCCTCAC
TATTGAAACTGTAGTTTTGCATAGTGTGGCTATATAA

5. Putative promoter region *AhSTS2*

TGCGGTTTGACGCGAGACCGCCCCAACACCATACAAAA

ACCGCGTATACGCGGATGTGGTCGTTCTTCGCTAGCAGCCTCCGCACCTCCACGTTG
AGG

CTGTCGTCAAGCTTCGCCACTAGTCGAGCACGAAGAAAGTGTGGATTTGAGACTGTA
GGT

GTAAGAGCTCCAACACAGTCTTCAGCAGCAAGCTCAGGAGCTTAATGATTATCGGG
AGAT

GTATAAGAAGAACCTCACCCGCATGACATCTACGGATGAGCTTAGGCTGGAGTGGA
GGGA

GTCGCTGGAGCGGATGCATCGTATGGAGGCTCAGATGGAGGTGTATCTGACCTAGA
TGCG

CGCCATTGGCATCGACCTTGCTAGTAGCAGCATCGCTGTTGGTGGCAGCAGCGATCT
TGT

TGGTGGCATATAGATATCACCGCCTTCTGCGCCGTCAACTCAGGGCCACGGGACTGA
CGA

CAAAGACGACGATTATCTGGATCTATAGTTATTAGTTTTTTGTTTTATTATTTCATTA
TA

TTTATTTGATGTACTTGACTTTCAATTTATTTGCACATTGTATTATTTATTTTAAATAA
A

AATTTTTTATTATTTATAAATTAATCACTAATTGTGTAAAAAAAATTTAAAATTAAAA
TT

AAAATTGATCATTTATTTCAAATTTAAAAAAATTGATATTACCGTTAAAGTAATCAT
AGG

AATAATTCGACGGTATTAGTGCGCCAATAATAGCGGTAGAAAAATTTGCCGGTAATC
AAT

TGATTTTTCGAGCTAACAGTAAATATTTACCAGTAAAATTTATACCGTCTAATTATTT
TT

TTATAGTAATTAATTTATCATTGAATTTTATTTATTTTTTCGATAATAAATTTAACGGT
A

CTTAACATTTTTTTCGTAATGTATGACTACTTATAATACTTATTATTTTTATATTATTT
A

TTATGTATATACATTATTTTATTTTGTTATTATAAAACAGACTGTGTTATTCAACAAC
AC

ACATGACATGATTATTGTTTGAAATAACAATATTTTCCTCCTTAATTAGCGATTTTAA
AT

TTGATTTTAATGTTATTCTTAAAAAAACCATTCGATGCACATATATTTCTATGTTAAC
AT

ATATGGCTCAACAAAAAATCACACTTAAAAGAGGTAATATATAGACCATTCAATTTG
ATC

ATTATTTTGGTGTCAATTCAATTTGACAATTGCCTAAATGGATGACTATTCCACATTT
TG

AATCCATGATTTTGAGAAGTTCATACGGAAACAATTAAGTCCTCCCTTTCTCATCAA
TTT

AAACACGAAAAAAAAGGCATTCTATAATTGTTCTTGGAAATATTACATTATGACATG
GAA

ATAATGGTAAGGTAGCACGTGAAACTCAGCTACCCACCTATCCATAAATCTTTCGTT
ACA

ATGGTGCATATAAAACCCCATCTTCCACTAACAAGTGAGCAACACAACACACCCTTT
AAT

TATTAGCTACTGCATTTCCTTC

Web link

file:///C:/Users/Lenovo%20330/AppData/Local/Temp/Temp1_PlantCARE_9139_Ahsts2_plantCARE.tar.zip/PlantCARE_9139_plantcare.html

6. Arachis hypogaea Stilbene synthase (*AhSTS3*) complete CDS

ATGGTGTCTGTGAGTGAGATCCGCAACATTCAAAGGGCAGAAGGCCCTGCAACTGT
ATTGGCAATTGGCACGGCAAATCCATCAAATTGTGTTGATCAGAGTACATATGCTGA
TTACTATTTCAGAGTAACCGATAGCGAACACATGACTGATCTCAAAAAGAAATTTCA

GCGCATTTGTGAAAGAACACAGATCAAGAACAGACATATGTACTTAACGGAAGAGA
TACTGAAAGAGAATCCTAACATGTGTGCATACAAGGCACCATCGTTGGATGCAAGG
GAAGATATGATGATTAGGGAGGTACCAAGGGTTGGAAAAGAGGCAGCAACCAAGG
CCATCAAAGAATGGGGACAGCCAATGTCTAAAATCACACATTTGATCTTCTGCACCA
CCAGCGGTGTTGCGTTGCCTGGCGTTGATTACGAACTCATCGTACTCTTAGGGCTCG
ACCCATGCGTCAAGAGGTACATGATGTACCACCAAGGCTGCTTCGCTGGCGGCACT
GTTCTTCGTTTGGCTAAGGACTTGGCTGAAAATAACAAGGATGCTCGTGTTCTTATT
GTTTGTTCTGAGAATACCGCAGTCACTTTCCGTGGTCCTAGTGAGACAGACATGGAT
AGTCTTGTAGGACAAGCATTATTTGCGGATGGAGCTGCTGCGATTATCATTGGTTCT
GATCCTGTGCCAGAGGTTGAGAAGCCTATCTTTGAGATTGTTTCGACTGATCAAAAG
CTCGTCCCTGGCAGCCATGGAGCTATCGGTGGTCTCCTTCGTGAAGTTGGGCTTACA
TTCTATCTTAACAAGAGTGTTCCTGATATTATTTCGCAAAATATCAACGACGCACTC
AGTAAAGCTTTTGATCCATTGGGTATATCTGATTATAACTCAATATTTTGGATTGCAC
ACCCTGGTGGACGTGCAATTTTGGACCAGGTTGAACAGAAGGTGAACTTGAAACCA
GAAAAAATGAAAGCTACTAGAGATGTGCTTAGTAATTATGGTAATATGTCAAGTGC
ATGTGTGTTCTTCATCATGGATTTGATGAGGAAGAAGTCTCTTGAAGAAGGACTTAA
AACCACCGGTGAAGGACTTGATTGGGGTGTGCTTTTTGGCTTTGGTCCTGGTCTCAC
CATTGAAACTGTTGTTCTTCGCAGTGTGACCATCTGA

7. Putative promoter region *AhSTS3*

ACTTAAAACATTTTATATTTGGATATTTTCATTTGTTATTTAGATTTTGAATTTGTATT
T

TAGTTACGACCTATCCCTCTTACACACACACTAGAGGCGATGTATTATTGCATGCAT
GCT

GATATAGACACTACTGGAATATCTTAAACAAATGTCTTATTGCAATATTATTGTTTA
AAA

GCATTGTTTTATTAGAAAAATGTTATTTAGACGAGAAGTCATCAATTTTTAATTAGTC
TT

TAAATTTAATAAATTAATTACATTAATTTTTATAAACTCACTTTTCAATTTTTTAACA
AA

CTAACACTGTTAGATTTAATTTAACTTATATTTCTAAAATCCCTCTTTTTTTCAAAATT
C

TCTATTTTCACCAAGTCTGATTCTTTACTCTTCTTGTACTGCTTTTTTTCCCCCTTAAA
A

TTTATACTTTATTATAAAGTATCTACCTAATAATAAATATAAAATAAAATAGTTAATT
AT

GTTATTGTTTAATTTGGTCAAGCATGATGACTAATTAATTAAGAACATACTCATATG
GTG

GTAGACTCCATTGTCATCCACCAACCTCCTCACACCCCCAGTCAAACTACATGTTCA
CTT

TGAATAATGAAATTTATTAAAAATGATAAGATCATAAATATACAATAAGTACCACA
AGTT

AATTAATCAATAACACACGAACATTTTTGCTTCGAAGAGATTTCAACATGACATGGA
CAA

AAATGCTACTAATGAAGCACGTGATGCCCAGCTACCTACATCTATTCAAAGGTAATT
ATT

ATAGTGTTTAAAAAATAGTATTTAATTTATAGAAAAAAAAGTTAAAAATTAATATTT
AAT

TTAAAAATAAAAGAATAAATAATTTTTAAATATTTAAAATTTATTATAAAAATTAAA
CAA

AAATTAGACACACTGTAAAATTGATCCTGTTCAAAATTTCAAATAGCCGTCACATAT
AAA

ATCTTATCTTCTAATAACAATTCTGAGATAGCTTGCTATAGTATGATTCGATGCATTA
GA

ACGATCAAATTAAAAGAAAGGAAAATTTTCGCAAACAAATAAAAATAAAACACACA
TTAA

AATAACAAATACTATATTCTACGATGAACAAACAAATAACAAATTCTATATTCTGGG
CAA

GCACAAAACAGAGATACATAAATCACTAAGAAGTCAGAATATCTGAGCCGCCATCC
ATAA

TAATAATATCATGAATTAATACTAAAACTACACGAATATATATATTCATGAACAATC
CAA

GTCAAAGTAACACAATAAAACAACCAAGAAGTCAGAATACATCAGCTGATCGCTTG
AATC

CAGGAACATTATTACGAGTAGTTTATTATACTTTGAGTTGAGTACGTTTCCTACATAT
GT

TTCAATTTGTCCATTTAATTCCACATATGCCCCTACTCTCATGAGTATAAATATCCCC
CC

TCCCCCTGCCTAAGTCTTCACCAAAATACCAAAGAACATAGCTAATAAGCTTTGCTA
TCC

C

Web link

file:///C:/Users/Lenovo%20330/AppData/Local/Temp/Temp1_PlantCARE_9798_Ahsts3_plantCARE.tar.zip/PlantCARE_9798_plantcare.html

8. Arachis hypogaea Stilbene synthase (*AhSTS4*) complete CDS

ATGGTGTCTGCAAGTGACATCCGCAAGGTTCAAAGAGCAGAAGGTCCTGCAACCGT
CTTAGCGATTGGCACAGCAAATCCACCAAACTGTGTTGATCAGAGCACATACGCAG
ATTACTATTTTAGAGTAACTAATAGCGAGCACATGACCGACCTCAAGAAGAAATTTC
AGCGCATTTGTGAGAGAACACAGATCAAGAACAGACATATGTATCTAACGGAAGAA
ATACTGAAGGAGAATCCTAACATGTGCGCATATAAAGCACCGTCCTTGGATGCAAG
GGAAGACATGATGATCAGGGAGGTACCAAGGGTTGGAAAAGAGGCTGCAACTAAG
GCAATCAAGGAATGGGGTCAGCCAATGTCTAAGATCACACATTTGATCTTCTGCACC
ACCAGCGGTGTTGCGTTGCCTGGCGTTGATTACGAACTCATCGTACTCTTAGGGCTC
GACCCAAGCGTCAAGAGGTACATGATGTACCACCAAGGCTGCTTCGCTGGCGGCAC
TGTCCTTCGTTTGGCTAAGGACTTGGCTGAAAACAACAAGGATGCTCGTGTGCTTAT
TGTTTGTTCTGAAAATACTTCAGTCACTTTTCGTGGTCCTAGTGAGACAGACATGGAT
AGTCTTGTAGGGCAAGCATTGTTTGCCGATGGAGCTGCTGCGATTATCATTGGTTCT
GATCCTGTTCCAGAGGTTGAGAATCCTCTCTTTGAGATTGTTTCAACTGATCAACAA
CTTGTCCCTAACAGCCATGGAGCCATCGGTGGTCTCCTTCGTGAAGTTGGACTTACA
TTCTATCTAACAAGAGTGTTCCGGATATTATTTCACAAAACATCAATGATGCACTCA
GTAAAGCTTTTGATCCACTAG

9. Putative promoter region *AhSTS4*

AATGATTGATGAGAAAGTTTTACTTAAAACATTTTATATTTGGATATTTTCATTTGTT
AT

TTAGATTTTGAATTTGTATTTTAGTTACGACCTATCCCTCTTACACACACACTAGAGG
CG

ATGTATTATTGCATGCATGCTGATATAGACACTACTGGAATATCTTAAACAAATGTC
TTA

TTGCAATATTATTGTTTAAAAGCATTGTTTTATTAGAAAAATGTTATTTAGACGAGA
AGT

CATCAATTTTTAATTAGTCTTTAAATTTAATAAATTAATTACATTAATTTTTATAAAC
TC

ACTTTTCAATTTTTTAACAAACTAACACTGTTAGATTTAATTTAACTTATATTTCTAA
AA

TCCCTCTTTTTTTCAAAATTCTCTATTTTCACCAAGTCTGATTCTTTACTCTTCTTGTA
C

TGCTTTTTTTCCCCCTTAAAATTTATACTTTATTATAAAGTATCTACCTAATAATAAA
TA

TAAAATAAAATAGTTAATTATGTTATTGTTTAATTTGGTCAAGCATGATGACTAATT
AAT

TAAGAACATACTCATATGGTGGTAGACTCCATTGTCATCCACCAACCTCCTCACACC
CCC

AGTCAAACTACATGTTCACTTTGAATAATGAAATTTATTAAAAATGATAAGATCATA
AAT

ATACAATAAGTACCACAAGTTAATTAATCAATAACACACGAACATTTTTGCTTCGAA
GAG

ATTTCAACATGACATGGACAAAAATGCTACTAATGAAGCACGTGATGCCCAGCTAC
CTAC

ATCTATTCAAAGGTAATTATTATAGTGTTTAAAAAATAGTATTTAATTTATAGAAAA
AAA

AGTTAAAAATTAATATTTAATTTAAAAATAAAAGAATAAATAATTTTTAAATATTTA
AAA

TTTATTATAAAAATTAAACAAAAATTAGACACACTGTAAAATTGATCCTGTTCAAAA
TTT

CAAATAGCCGTCACATATAAAATCTTATCTTCTAATAACAATTCTGAGATAGCTTGC
TAT

AGTATGATTCGATGCATTAGAACGATCAAATTAAAAGAAAGGAAAATTTTCGCAAA
CAAA

TAAAAATAAAACACACATTAAAATAACAAATACTATATTCTACGATGAACAAACAA
ATAA

CAAATTCTATATTCTGGGCAAGCACAAAACAGAGATACATAAATCACTAAGAAGTC
AGAA

TATCTGAGCCGCCATCCATAATAATAATATCATGAATTAATACTAAAACTACACGAA
TAT

ATATATTCATGAACAATCCAAGTCAAAGTAACACAATAAAACAACCAAGAAGTCAG
AATA

CATCAGCTGATCGCTTGAATCCAGGAACATTATTACGAGTAGTTTATTATACTTTGA
GTT

GAGTACGTTTCCTACATATGTTTCAATTTGTCCATTTAATTCCACATATGCCCCTACT
CT

CATGAGTATAAATATCCCCCCTCCCCCTGCCTAAGTCTTCACCAAAATACCAAAGAA
CATA

Web link

file:///C:/Users/Lenovo%20330/AppData/Local/Temp/Temp1_PlantCARE_10083_Ahsts4_plantCARE.tar.zip/PlantCARE_10083_plantcare.html

10. Arachis hypogaea Stilbene synthase (*AhSTS6*) complete CDS

ATGGTGTCTGCGAGTGACATCCGCAAAGTTCAAAGAGCAGAAGGCCCTGCAACTGT
ATTAGCGATTGGCACAGCAAATCCACCAAACTGTGTTGATCAGAGCACATATGCAG
ATTACTATTTTAGAGTAACTAATAGCGAGCACATGACCGACCTCAAGAAAAAATTTC
AGCGCATTTGTGAGAGAACACAGATCAAAAACAGATATATGTATTTAACGGAAGAA
ATACTAAAGGAGAATCCTAACATGTGCGCATACAAAGCACCGTCCTTGGATGCAAG
AGAAGACATGATGATCAGGGAAGTACCAAGGGTTGGAAAAGAGGCTGCAACTAAG
GCAATCAAGGAATGGGGTCAGCCAATGTCTAAAATCACACATTTGATCTTCTGCACC
ACCAGCGGCGTTGCATTGCCTGGCGTTGATTATGAACTCATCGTACTCTTAGGGCTT
GACCCAAGCGTCAAGAGGTACATGATGTACCACCAAGGCTGCTTTGCTGGCGGCAC
TGTCCTTCGTTTGGCTAAGGACTTGGCTGAAAACAACAAGGATGCTCGTGTGCTTAT
TGTTTGTTCTGAAAATACTTCAATCACCTTTCGTGGTCCTAGTGAGACAGACATGGA
TAGTCTTGTAGGGCAAGCATTGTTTGCTGATGGAGCTGCTGCGATTATCATTGGTTCT
GATCCTATTCCAGATGTTGAGAATCCTCTCTTTGAGATTGTTTCAACTGATCAACAAC
TTGTCCCTAACAGCCATGGAGCCATCGGTGGTCTCCTTCGTGAAGTTGGACTTACAT
TCTATCTTAACAAGAGTGTTCCGGATATTATTTCACAAAACATCAATGATGCACTCA
CTAAAGCTTTTGATCCACTAGGTATATCTGATTATAACTCAATATTTTGGATTGCACA
TCCTGGTGGACGTGCAATTTTGGACCAAGTTGAAGAGAAGGTGAACTTGAAACCAG
AGAAGATGAAAGCCACTAGAGATGTGCTTAGCAATTATGGTAACATGTCAAGTGCA
TGTGTGTTCTTCATTATGGATTTGA

11. Putative promoter region *AhSTS6*

TTGAATTTTAATGATTGATGAGAAAGTTTTACTTAAAACATTTTATATTTGGATATTT
TC

ATTTGTTATTTAGATTTTGAATTTGTATTTTAGTTACGACCTATCCCTCTTACACACAC
A

CTAGAGGCGATGTATTATTGCATGCATGCTGATATAGACACTACTGGAATATCTTAA
ACA

AATGTCTTATTGCAATATTATTGTTTAAAAGCATTGTTTTATTAGAAAAATGTTATTT
AG

ACGAGAAGTCATCAATTTTTAATTAGTCTTTAAATTTAATAAATTAATTACATTAATT
TT

TATAAACTCACTTTTCAATTTTTTAACAAACTAACACTGTTAGATTTAATTTAACTTA
TA

TTTCTAAAATCCCTCTTTTTTTCAAAATTCTCTATTTTCACCAAGTCTGATTCTTTACT
C

TTCTTGTACTGCTTTTTTTCCCCCTTAAAATTTATACTTTATTATAAAGTATCTACCTA
A

TAATAAATATAAAATAAAATAGTTAATTATGTTATTGTTTAATTTGGTCAAGCATGA
TGA

CTAATTAATTAAGAACATACTCATATGGTGGTAGACTCCATTGTCATCCACCAACCT
CCT

CACACCCCCAGTCAAACTACATGTTCACTTTGAATAATGAAATTTATTAAAAATGAT
AAG

ATCATAAATATACAATAAGTACCACAAGTTAATTAATCAATAACACACGAACATTTT
TGC

TTCGAAGAGATTTCAACATGACATGGACAAAAATGCTACTAATGAAGCACGTGATG
CCCA

GCTACCTACATCTATTCAAAGGTAATTATTATAGTGTTTAAAAAATAGTATTTAATTT
AT

AGAAAAAAAAGTTAAAAATTAATATTTAATTTAAAAATAAAAGAATAAATAATTTT
TAAA

TATTTAAAATTTATTATAAAAATTAAACAAAAATTAGACACACTGTAAAATTGATCC
TGT

TCAAAATTTCAAATAGCCGTCACATATAAAATCTTATCTTCTAATAACAATTCTGAG
ATA

GCTTGCTATAGTATGATTCGATGCATTAGAACGATCAAATTAAAAGAAAGGAAAAT
TTTC

GCAAACAAATAAAAATAAAACACACATTAAAATAACAAATACTATATTCTACGATG
AACA

AACAAATAACAAATTCTATATTCTGGGCAAGCACAAAACAGAGATACATAAATCAC
TAAG

AAGTCAGAATATCTGAGCCGCCATCCATAATAATAATATCATGAATTAATACTAAAA
CTA

CACGAATATATATATTCATGAACAATCCAAGTCAAAGTAACACAATAAAACAACCA
AGAA

GTCAGAATACATCAGCTGATCGCTTGAATCCAGGAACATTATTACGAGTAGTTTATT
ATA

CTTTGAGTTGAGTACGTTTCCTACATATGTTTCAATTTGTCCATTTAATTCCACATAT
GC

CCCTACTCTCATGAGTATAAATATCCCCCCTCCCCCTGCCTAAGTCTTCACCAAAATACC

Web link

file:///C:/Users/Lenovo%20330/AppData/Local/Temp/Temp1_PlantCARE_9636_Ahsts6_plantCARE.tar.zip/PlantCARE_9636_plantcare.html
